# Automated CT Lung Density Analysis of Viral Pneumonia and Healthy Lungs Using Deep Learning-Based Segmentation, Histograms and HU Thresholds

**DOI:** 10.3390/diagnostics11050738

**Published:** 2021-04-21

**Authors:** Andrej Romanov, Michael Bach, Shan Yang, Fabian C. Franzeck, Gregor Sommer, Constantin Anastasopoulos, Jens Bremerich, Bram Stieltjes, Thomas Weikert, Alexander Walter Sauter

**Affiliations:** 1 Department of Radiology, University Hospital Basel, University of Basel, Petersgraben 4, 4031 Basel, Switzerland; andrej.romanov@usb.ch (A.R.); gregor.sommer@usb.ch (G.S.); jens.bremerich@usb.ch (J.B.); thomas.weikert@usb.ch (T.W.); alexander.sauter@usb.ch (A.W.S.); 2 Department of Research & Analytic Services, University Hospital Basel, University of Basel, Spitalstrasse 8, 4031 Basel, Switzerland; michael.bach@usb.ch (M.B.); shan.yang@usb.ch (S.Y.); Fabian.Franzeck@usb.ch (F.C.F.); bram.stieltjes@usb.ch (B.S.)

**Keywords:** viral pneumonia, histogram analysis, imaging biomarker, computed tomography, artificial intelligence

## Abstract

CT patterns of viral pneumonia are usually only qualitatively described in radiology reports. Artificial intelligence enables automated and reliable segmentation of lungs with chest CT. Based on this, the purpose of this study was to derive meaningful imaging biomarkers reflecting CT patterns of viral pneumonia and assess their potential to discriminate between healthy lungs and lungs with viral pneumonia. This study used non-enhanced and CT pulmonary angiograms (CTPAs) of healthy lungs and viral pneumonia (SARS-CoV-2, influenza A/B) identified by radiology reports and RT-PCR results. After deep learning segmentation of the lungs, histogram-based and threshold-based analyses of lung attenuation were performed and compared. The derived imaging biomarkers were correlated with parameters of clinical and biochemical severity (modified WHO severity scale; c-reactive protein). For non-enhanced CTs (*n* = 526), all imaging biomarkers significantly differed between healthy lungs and lungs with viral pneumonia (all *p* < 0.001), a finding that was not reproduced for CTPAs (*n* = 504). Standard deviation (histogram-derived) and relative high attenuation area [600–0 HU] (HU-thresholding) differed most. The strongest correlation with disease severity was found for absolute high attenuation area [600–0 HU] (r = 0.56, 95% CI = 0.46–0.64). Deep-learning segmentation-based histogram and HU threshold analysis could be deployed in chest CT evaluation for the differentiating of healthy lungs from AP lungs.

## 1. Introduction

Recent advances in machine learning (ML) enable a swift extraction of imaging biomarkers from chest CT scans [1]. Automated approaches are especially desirable during busy periods for radiology departments, such as during the current public health crisis related to SARS-CoV-2, and are more reproducible compared to approaches that require user interaction [2,3,4,5,6,7,8,9,10]. A prerequisite for the analysis of pulmonary imaging biomarkers is a reliable automated segmentation of the lung, which has become feasible [11,12,13].

Regarding density analysis of lung disease in CT, histograms are a robust method for the visualization and quantification of Hounsfield unit (HU) differences [14]. Shifts in density histogram curves have been used to differentiate between normal lungs and those affected by structural lung pathologies, such as fibrosis and emphysema [15,16,17,18,19]. Furthermore, a variety of HU thresholds have been proposed to identify pathologic changes in lungs [11,20,21,22]. For example, a HU threshold of <−950 HU became a convention for quantification of emphysema with CT images [23], whereas commonly accepted standards for other lung pathologies are lacking.

Histogram analysis and HU thresholding have very rarely been applied to analyzing viral pneumonia and its radiological pattern of atypical pneumonia (AP). Pulmonary changes in atypical pneumonia particularly manifest as areas of increased density (high attenuating areas (HAA)), caused by ground glass opacities (GGO) and consolidations [24,25,26,27,28,29].

In non-immunocompromised patients, AP is often caused by influenza A and B viruses [24]. During the current pandemic, SARS-CoV-2 (severe acute respiratory syndrome coronavirus 2) has become another frequent cause of AP [30]. The chest CT plays a major role in the diagnostic workup of suspected pulmonary manifestations of viral disease and associated complications. Radiology reports usually describe them qualitatively [26,31]. However, there is a huge clinical demand for quantitative imaging biomarkers of pulmonary alterations. How these imaging biomarkers can add established biochemical and clinical markers such as c-reactive protein (CRP) and the need for oxygen therapy [10,32,33,34,35,36,37,38,39] remains an open question.

Additionally, administration of contrast agents has a major influence on quantitative chest CT analysis and therefore should be considered [40]. Non-enhanced computed tomography (NECT) and the computed tomography pulmonary angiogram (CTPA) are important protocols in chest imaging. The latter is especially relevant for the evaluation of diseases with procoagulant properties, such as coronavirus disease 2019 (COVID-19) and influenza [41,42,43].

This study had three main goals. First, to automatically derive imaging biomarkers from chest CT images based on histogram and HU-threshold analysis. Second, to assess differences in those biomarkers between lung-healthy individuals vs. those affected by atypical pneumonia. Third, to correlate the imaging biomarkers with biochemical and clinical severity, defined by CRP and a modified World Health Organization (WHO) clinical severity scale [44].

## 2. Materials and Methods

### 2.1. Study Population

#### 2.1.1. Healthy Lung Group

**Figure 1:**
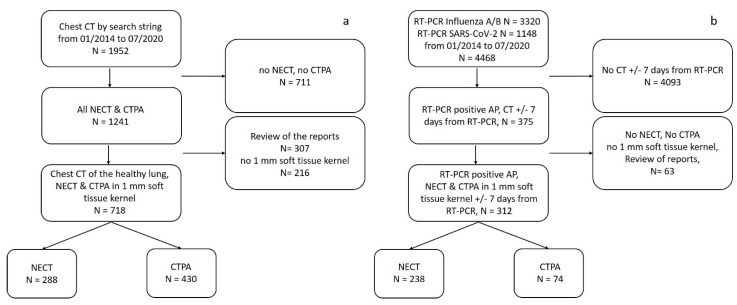
Study selection flow diagrams for (**a**) healthy lung and (**b**) AP groups. Abbreviations: NECT = non-enhanced computed tomography. CTPA = computed tomography pulmonary angiogram. AP = atypical Pneumonia.

All chest CTs of healthy lungs acquired at our institution between January 2014 and July 2020 were identified by an appropriate text search of the radiology reports, which were structured (search string: unremarkable lung parenchyma AND no pulmonary mass AND open central airways AND no pleural effusion AND no pneumothorax) (Figure 1a). At least one board-certified radiologist had signed these reports. NECTs and CTPAs were selected based on study descriptions. Only studies with 1 mm soft-tissue kernel reconstructions were analyzed, because the lung segmentation algorithm is optimized for those image reconstruction parameters. Reports containing keywords that indicate non-normal lung architecture, such as “status post lung surgery” and “structural lung abnormalities” or describing motion artifacts were excluded to avoid bias at later stages of the analysis as suggested by Best et al. [18]. All reports were reviewed by a radiology resident in the first post-graduate year (A.R.).

#### 2.1.2. Atypical Pneumonia Group

All patients with a positive RT-PCR test for influenza A/B or SARS-CoV-2 were identified (time period: January 2014 and July 2020). The information was retrieved from the laboratory information system. For those cases, we searched the Picture Archiving and Communication System (PACS) to identify all NECTs and CTPAs performed up to seven days before or after the positive RT-PCR test (Figure 1b). Scans with motion artefacts and patients with “status post lung surgery” according to the written radiology report were excluded. The mean time interval in days between RT-PCR and CT acquisition was determined. Figure 1 displays the full selection workflow.

### 2.2. CT Imaging

CTs were acquired in supine position on the following CT scanners: SOMATOM Definition Edge, SOMATOM Definition AS+, SOMATOM Definition Flash, SOMATOM Force (all Siemens Healthineers, Forchheim, Germany). Detailed information on acquisition parameters is provided in Supplement S1.

### 2.3. Lung Segmentation Algorithm

All chest CTs underwent automated lung segmentation using 1 mm soft-tissue kernel series as input to a convolutional neural network with U-Net architecture. It had been trained on 172 chest CTs of the same institution (86 COVID-19 and 86 non-COVID-19). The algorithm performs excellently (DICE score: 0.97) and had been described in detail as “A2” in a previous publication [11].

### 2.4. Histogram Analysis

Based on CT lung segmentations, HU density histograms of the whole lungs were calculated, using Python (package: SciPy, version 1.5.4) [45]. A HU density range from −1024 to 0 HU was chosen for further analysis [46]. This range included the density distribution of normal lung structure as well as all HU thresholds analyzed in this study. Density histograms were plotted with an interval of 1 HU and normalized to an area under the histogram curve of 1. As imaging biomarkers, the following standard histogram parameters were calculated for each lung [15,16,17,18,19,40]: mean lung attenuation, median lung attenuation, histogram standard deviation, skewness and kurtosis. For comprehensive parameter definitions, please refer to Supplement S2.

### 2.5. HU Threshold Analysis

First mentioned in the context of diffuse interstitial lung disease by Lederer et al. [20], HAA is the lung volume (in mL) containing CT attenuation values higher than those of normal lung parenchyma. In healthy lungs, HAA is very low and reflects physiological structures such as vessels and bronchial walls [46]. In pneumonia, HAA additionally corresponds to superimposed disease-related alterations such as ground glass opacities (GGOs) and consolidations [24,25,26,27,28,29,30,47]. rHAA (in %) is the ratio of HAA over the total lung volume. 

Four thresholds for HAA quantification reported in previous studies were evaluated (lower/upper threshold): −600/0 HU [11], −600/−250 HU [20], −700/−251 HU [21] and −800/−500 HU [22]. Both HAA and rHAA were calculated for all four HU ranges.

### 2.6. Statistical Analysis

#### 2.6.1. General Information

Mean and standard deviation were used to describe continuous variables. To assess relationships of two categorical variables, the Chi^2^ test was used. Differences between two independent groups regarding normally distributed continuous variables were tested using a t-test, and for not normally distributed data, a Mann–Whitney U test was used; *p*-values of less than 0.05 were considered significant. Bonferroni correction was performed in cases of multiple comparisons.

Statistical calculations were performed with SPSS (IBM SPSS Statistics, version 25.0, 2018). Python was used for data analysis and visualization (libraries: SciPy [45]; pandas [48]; scikit-learn [49]; matplotlib [50]; seaborn [51]). For visualization of different HU thresholds MITK (v2018.04.2) was used [52].

#### 2.6.2. Differences in Imaging Biomarkers between Healthy Lungs and AP

Tests for significant differences of the imaging biomarkers between the healthy lung and the AP group were performed independently for NECTs and CTPAs.

To address the question of whether histogram and HU threshold analyses differ significantly between healthy lungs and those with AP, the mutual information classifier (MIC) was used. The MIC was used to rank each derived histogram parameter regarding its ability to differentiate between two groups and measured the degrees of dependency among variables from 0 to 1, with 1 meaning perfect dependency. In our analysis, the two variables were the metrics on the one hand and the group (normal vs. AP) on the other hand. The function relies on nonparametric methods based on entropy estimation from k-nearest neighbors’ distances as described in [53,54], and was implemented with SciPy.

#### 2.6.3. Correlation of Imaging Biomarkers with CRP and Clinical Severity in the AP Group

The blood inflammation marker CRP [32] on the day of CT acquisition was retrieved from the laboratory information system for patients in the AP group. The CRP was selected because (a) it is a standard parameter of inflammation and (b) is tested on an almost daily basis. In case of missing same-day laboratory values, the closest value in a time interval of 2 days was chosen. Furthermore, disease severity was graded according to a modified, five-point ordinal scale for clinical severity published by the WHO [44]. This scale was based on the patient’s oxygen demand and ventilation status (for details, see Supplement S3). To determine correlation of histogram parameters and HAA/rHAA with CRP and disease severity, the Spearman rank correlation was used.

## 3. Results

### 3.1. Patient Characteristics

In total, 1030 CT studies were analyzed (50.9% women; mean age ± SD: 54.2 years ± 18.9). NECTs (*n* = 526) were performed for 288 healthy lung patients (40.6% women; mean age: 50.2 years ± 17.0) and 238 AP patients (35.3% women; mean age: 60.1 years ± 17.2), whereas CTPAs (*n* = 504) were performed for 430 healthy lung patients (65.8% women; mean age: 51.6 years ± 19.9) and 74 AP patients (54.1% women; mean age: 66.0 years ± 16.0). For NECT and CTPA, age differed significantly between the two groups, but sex did not. CT scans for AP were requested on average 1.9 days ± 2.3 days around RT-PCR-testing (influenza A/B ± SD: 1.6 days ± 2.2 days; COVID-19 ± SD: 2.1 days ± 2.4 days). Mean lung volume was 4475.9 mL ± 1270.9 mL. For detailed information, please refer to Table 1.

### 3.2. Discrimination of Healthy Lungs andAtypical Pneumonia: NECT and CTPA

Altogether, 526 NECTs and 504 CTPAs were analyzed. Regarding NECTs, all parameters derived from histograms and HU thresholds differed significantly between the healthy lung group and the AP group (*p*-value < 0.001). This finding persisted after correction for multiple testing (α < 0.05/13 ≈ 0.0038). In detail, the AP group showed higher histogram standard deviation, higher mean lung attenuation, higher median lung attenuation, lower skewness and lower kurtosis (all *p*-values < 0.001). HAA and rHAA were higher in the AP group compared to the healthy lung group (all *p*-values < 0.001) (Table 2).

**Figure 2:**
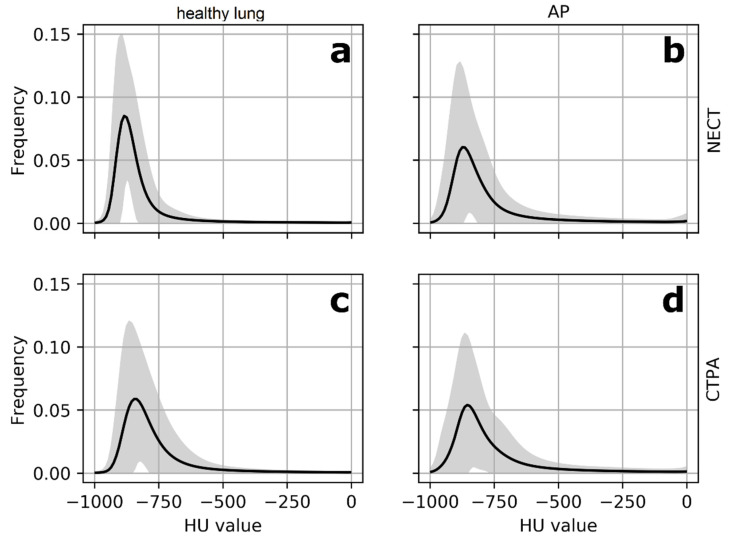
Mean histograms for NECT and CTPA. Mean histograms (black line) for NECTs (**a**,**b**) and CTPAs (**c**,**d**) with grey areas indicating variance (2SD). HU values on the *x*-axis, frequency on the *y*-axis. The differences in mean histogram curves between the healthy lung group and the AP group are significant for NECTs (**a**,**b**) regarding all histogram parameters and HAA/rHAA. Differences for CTPAs are subtle (**c**,**d**). Abbreviations: NECT = non-enhanced computed tomography. CTPA = computed tomography pulmonary angiogram. AP = atypical pneumonia. HAA = high attenuation area. rHAA = relative high attenuation area. SD = standard deviation.

In CTPAs, differences between the study groups were not significant different for a third of the parameters (*p*-values: 0.185–0.842). In general, group differences were much smaller in CTPAs compared to NECTs (Table 2). Figure 2 illustrates this observation. Therefore, the analysis was continued for NECT only.

### 3.3. Differences in Imaging Biomarkers between Healthy Lung and AP Group

**Figure 3:**
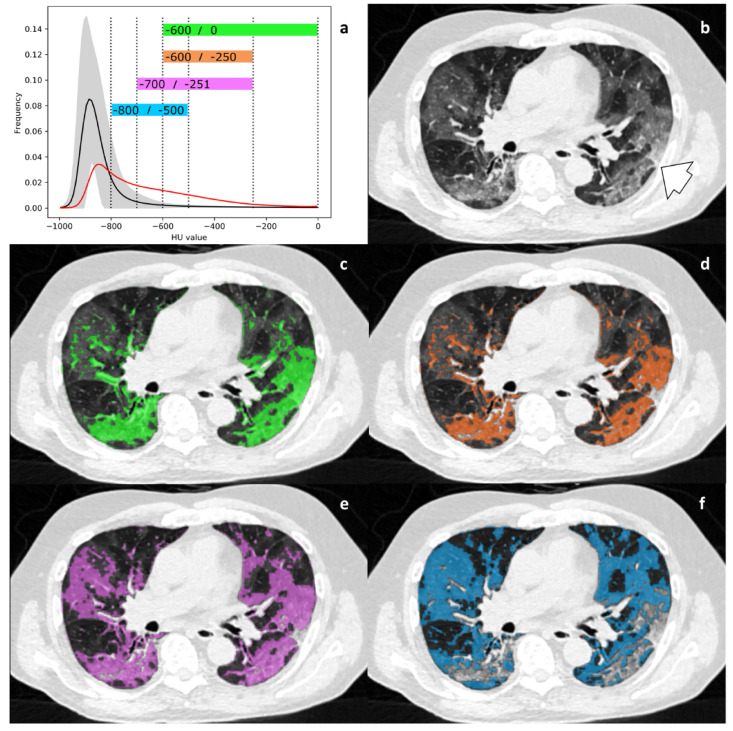
(**a**–**f**). Mean histogram and HU thresholds. Mean histogram curve of the healthy control group with all analyzed HU thresholds (a; dotted lines; black line = mean, grey area = 2 SD). The red curve indicates the histogram of a 75-year-old patient with diagnosed COVID-19. Note the overlap with the mean histogram curve of the healthy lung group below −750 HU. Within the range of −750 to −600 HU, HAA in healthy lungs start to diminish, whereas in this representative case of AP, HAA remains high. Best discriminatory power is seen in HAA-600/-250 and HAA-600/0, respectively, as HAA in AP is high and HAA in healthy lungs approaches zero. Figure 3b shows the corresponding chest CT. Bilateral and peripheral GGO is noted, but so is subtle consolidation in the posterior segment of the right upper lobe (arrow). Four HU threshold-derived HAAs (colored boxes in Figure 3a) are shown as overlays in Figure 3c–f. GGOs and consolidations are included according to their density. HAA −800/−500 only captures mild GGOs; HAA-600/0 also includes denser consolidation. Abbreviations: AP = atypical pneumonia. HAA = high attenuation area. GGO = ground glass opacity. SD = standard deviation.

**Figure 4:**
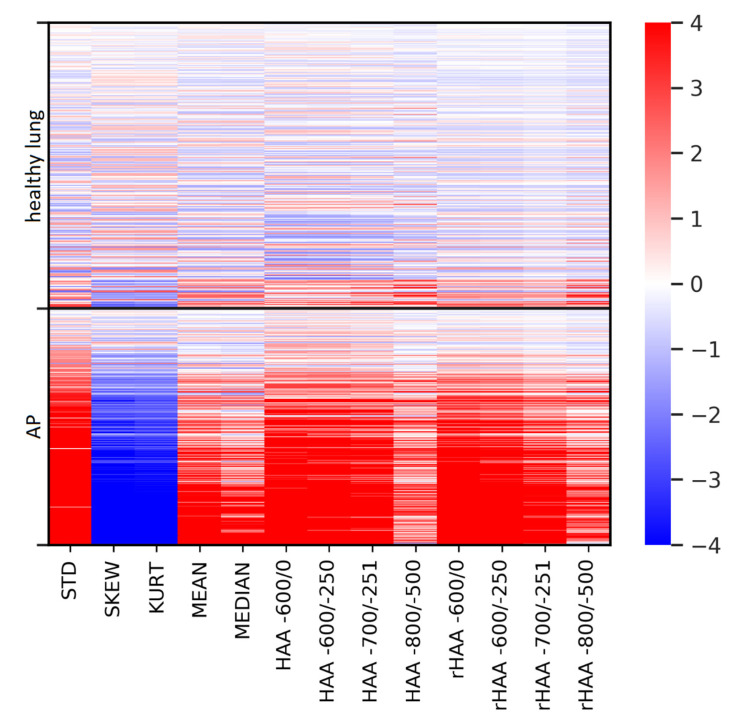
Imaging parameters for differentiation between healthy lungs and AP. An overview of all cases included in the analysis illustrating the differences in imaging biomarkers between the healthy lung group and the AP group. Color intensity represents the deviation of the imaging biomarker from the mean of the “healthy lung” group (in SD). Abbreviations: AP = atypical pneumonia. NECT = non-enhanced computed tomography. STD = histogram standard deviation. SKEW = skewness. KURT = kurtosis. MEAN = mean lung attenuation. MEDIAN = median lung attenuation. HAA = high attenuation area. rHAA = relative high attenuation area. SD = standard deviation.

The parameter histogram standard deviation (MIC: 0.37) and the HU threshold rHAA-600/0 (MIC: 0.35) differed most clearly between healthy controls and AP. Their high MIC reflects the high dependency of the imaging biomarkers on the group membership healthy vs. AP. Low MIC in this context means that the values of an imaging biomarker are independent from group membership, rendering it not useful to separate those two groups. Table 3 provides details. Figure 3 provides an exemplary histogram and the four HAAs for a 75-year-old patient with RT-PCR confirmed COVID-19. Figure 4 provides an overview over the difference of all imaging parameters of all cases compared to the mean “healthy lung” histogram.

### 3.4. Correlation of Parameters with CRP and the Clinical Severity Scale in the AP Group

The mean time interval between acquisition of CT scans and RT-PCR was 1.9 days ± 2.3. The mean CRP was 68.93 mg/L ± 74.11; the mean severity scoring according to the modified WHO scale was 2.38 ± 0.93.

**Figure 5:**
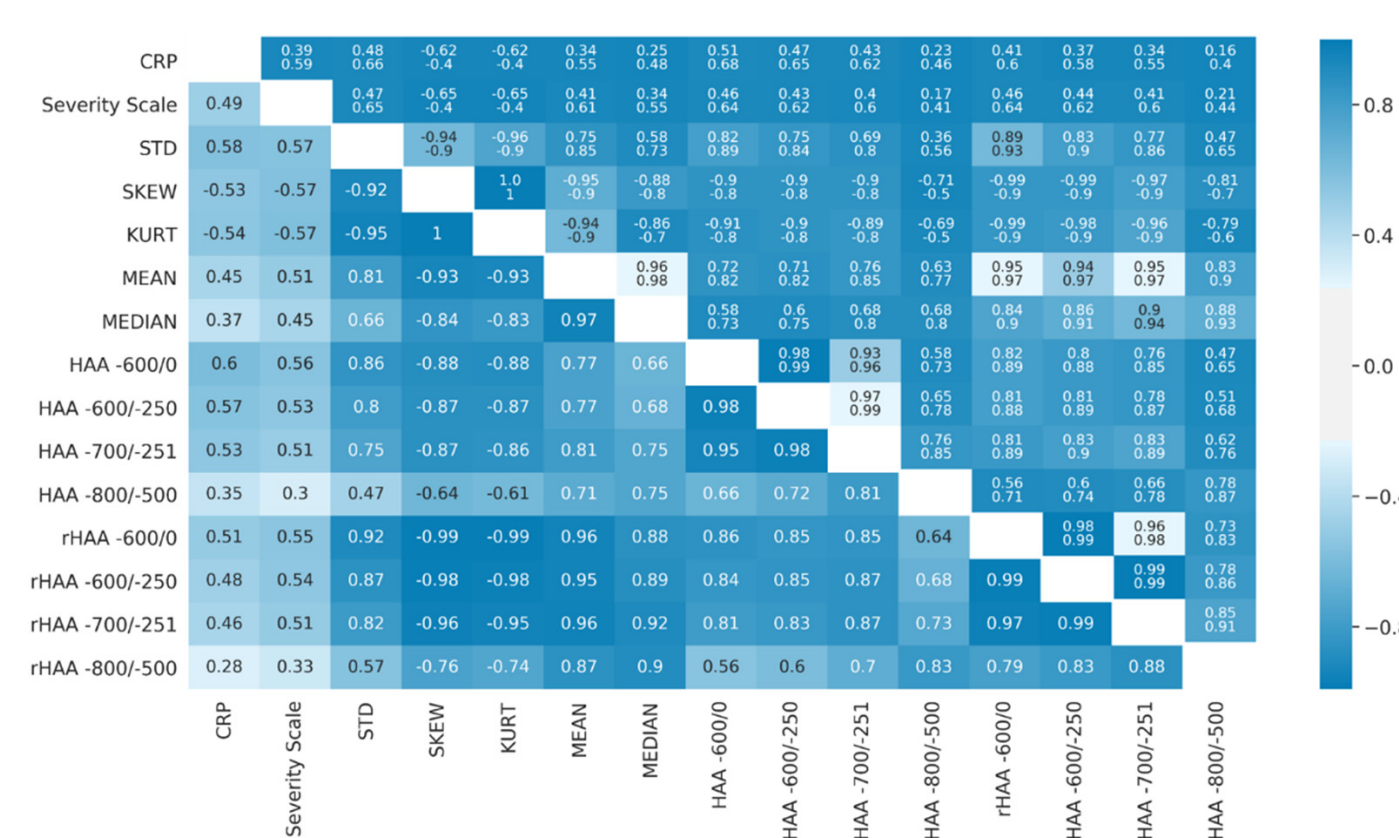
Spearman rank correlations (lower triangle) and 95% confidence intervals (upper triangle, with the lower confidence interval in the first row and the upper in the second row, respectively) of imaging biomarkers with clinical parameters (CRP and clinical severity scale). The colormap applies to the Spearman rank correlation values and the range of confidence intervals. Abbreviations: NECT = non-enhanced computed tomography. STD = histogram standard deviation. SKEW = skewness. KURT = kurtosis. MEAN = mean lung attenuation. MEDIAN = median lung attenuation. HAA = high attenuation area. rHAA = relative high attenuation area.

The correlation coefficients of imaging biomarkers with CRP ranged from r = |0.28| to |0.60| and the clinical severity score from |0.30| to |0.57|. The highest correlations with CRP regarding the two approaches of density analysis were observed for histogram standard deviation (r = 0.58, 95%, CI = 0.48–0.66), HAA-600/0 (r = 0.60, 95% CI = 0.51–0.68) and rHAA-600/0 (r = 0.51, 95% CI = 0.41–0.60). The clinical severity scale showed highest correlations with histogram standard deviation (r = 0.57, 95% CI = 0.47–0.65), skewness (r = −0.57, 95% CI = 0.65–0.40) and kurtosis (r = −0.57, 95% CI = 0.65–0.40)—respectively, HAA-600/0 (r = 0.56, 95% CI = 0.46–0.64) and rHAA-600/0 (r = 0.55, 95% CI = 0.46–0.64). Figure 5 provides details, and Supplement S4 provides the coefficient of determination.

## 4. Discussion

This study analyzed two approaches to automated CT lung density analysis in patients with healthy lungs and RT-PCR-confirmed atypical pneumonia: histogram analysis and HU-thresholding. All analyses were based on an established, automated segmentation of the whole lungs using deep learning. The majority of the derived imaging biomarkers differed significantly between the healthy lung group and the AP group. However, the effect of contrast medium considerably weakened differences in CTPAs. On NECTs, the histogram-derived parameters histogram standard deviation and rHAA-600/0 showed the highest discriminatory potential between healthy lungs and lungs affected by AP. Furthermore, standard histogram-based parameters and threshold-based quantification showed strong correlations with CRP and a clinical scale for disease severity in AP, indicating clinical meaningfulness.

Obert et al. applied CT histogram analysis to differentiate normal lungs from those affected by emphysema and fibrotic changes [15]. The facts that the sample size was small and different pathologies were analyzed hampered a side-by-side comparison with the data of the study at hand. However, histograms of CTs with increasing degrees of fibrosis, a condition that results in areas with HAA, just like atypical pneumonia, showed a flattening of the curve and a shift to the right. This is also well reflected in the histograms of our AP group.

Ash et al. have examined associations between both local histogram-based quantitative CT measurements and densitometry with pulmonary function test parameters and mortality in 46 patients with idiopathic pulmonary fibrosis [16]. Among others, higher HAA, lower skewness and lower kurtosis were associated with worse survival. This points in the same direction as our study that identified high correlations of histogram parameters with CRP and a clinical severity scale. Their approach differed methodologically from the one used in our study. Furthermore, the authors did not correlate the extracted features with clinical or biochemical severity, nor did they assess different imaging protocols.

So far, studies assessing the manifestations of viral pneumonia by means of HU thresholds are rare. Four different studies evaluated the presence of GGOs in parenchymal lung disease and COVID-19 pneumonia. HAA and rHAA in healthy lungs are meant to reflect the baseline of normal lung structure without pathogenic components. This baseline needs to be considered in the interpretation of severity from HAA/rHAA in AP. In a previous study, Mascalchi et al. [55] emphasized the dependency of quantitative parameters and lung volume. Indeed, we found lower lung volumes in the AP group that underwent NECT compared to healthy lungs. We therefore propose to consider rHAA over HAA in the quantification of disease-related alterations in AP.

A preliminary study evaluated a fixed HU threshold for the quantification of pulmonary opacities in COVID-19 [11] and reported a HU-range of –600/0 HU to be suitable for quantification and segmentation of affected areas. Furthermore, [11] reported higher mean HAA-600/0 values in those studies with iodine contrast applied in comparison to NECTs, which is in line with our results and was probably due to a higher HAA-baseline caused by partial volume effects of peripheral pulmonary arteries. This could also explain the fact that normal lungs and those affected by AP were much better separable on NECT compared to CTPA. It is noteworthy that the HU threshold, which does not consider GGOs alone, provides the best classification and correlates best with clinical parameters. Hence, the type of GGOs influences severity stratification. One can assume that denser HAA such as in the HU range of −600/0 HU reflect the extent of disease more precisely than light GGOs alone in a HU range of −800/−500 HU.

Sumikawa et al. [21] used another HU threshold (−700/−251 HU) to differentiate usual interstitial pneumonia and nonspecific interstitial pneumonia in 60 patients. The numbers of voxels with higher density correlated with three parameters (contrast, variance and entropy). The authors concluded that volume histogram analysis for cubic ROIs may be feasible for differentiating between usual interstitial pneumonia and non-specific interstitial pneumonia. In our opinion, approaches using whole lung segmentation are more robust than manually defined ROIs. An inverse approach to HAA analysis was performed by Colombi et al. [56] with the quantification of well-aerated lung areas using a HU range of −950 to −700 HU in COVID-19. The authors found significant differences between CTs of patients requiring intensive care unit admission, which is consistent with the strong correlation of HAA with the modified WHO scale for severity found in our study. Similar observations were reported by Leonardi et al. [9] and Lanza et al. [10], showing the predictive potential of quantitative CT parameters for the need for oxygenation support and intubation in COVID-19.

To further study the clinical meaningfulness of CT features extracted from NECTs, correlations of imaging biomarkers with CRP were assessed. CRP is a standard laboratory parameter of inflammation and commonly elevated in patients with influenza and COVID-19 [33,38,39]. Sun et al. described a correlation of r = 0.49 between CRP and a total lesion CT score [3], which is consistent with our results (HAA-600/0: r = 0.60, 95% CI = 0.51–0.68).

This study has limitations. First, it was designed as a joint analysis of two important viral pathogens causing AP, influenza virus and SARS-CoV-2. In our opinion, the facts that the CT imaging phenotypes of various causes of AP resemble each other and that this analysis is based on imaging characteristics justify this approach. Second, the methods used aggregate information on the density of the whole lung. Information on location, which is diagnostically important, is therefore disregarded. However, the intention of this study was not to state a diagnosis for the underlying disease. Third, we are aware of the potential influence of acquisition and reconstruction parameters on our results. This being a single center study and scanners from one vendor being used may influence the reproducibility. On the other hand, image-derived parameters matched clinical parameters and were comparable to previous studies. A collection of sample datasets is available under Supplement S5. Fourth, the AP group contained the whole range of pulmonary imaging findings. This also included a small number of CTs without infiltrates. The fact that all imaging biomarker differences between the healthy and AP group were nonetheless significant for NECTs underlines the discriminatory potential of the two approaches. Fifth, CRP is not distinct for diagnosis of COVID-19; nevertheless, it is an established biochemical parameter for assessment of severity in infectious and inflammatory diseases. Sixth, the MIC is a measure of differing probability distributions and thus provides a group discriminatory index, yet is not suited to assign an examination to one of these groups.

## 5. Conclusions

To conclude, this study showed that both histogram analysis and HU thresholds are promising sources of automatically extractable meaningful imaging biomarkers. They differ significantly between NECTs of healthy lungs and those of patients affected by AP. These approaches seem less suited for analysis of CTPAs. Of note, histogram standard deviation and rHAA-600/0 were the parameters that differed most between the two groups. Furthermore, the derived imaging biomarkers correlate with CRP and a clinical severity scale, supporting their clinical meaningfulness. In our opinion, rHAA-600/0 is the most intuitive, comprehensible and comparable parameter and is therefore usable in clinical practice and for further studies.

## Figures and Tables

**Table 1:** Patient demographics.

	Total	NECT			CTPA		
		Healthy Lung	AP	*p*	Healthy Lung	AP	*p*
***n***	1030	288	238		430	74	
**Sex** **[F/M, F%]**	524/506, 50.9%	117/171, 40.6%	84/154, 35.3%	0.245	283/147, 65.8%	40/34, 54.1%	0.069
**Age [years/SD]**	54.2/18.9	50.2/17.0	60.1/17.2	<0.001	51.6/19.9	66.0/16.0	<0.001

Abbreviations: AP = atypical pneumonia. NECT = non-enhanced computed tomography. CTPA = computed tomography pulmonary angiogram. SD = standard deviation. *p* = *p*-value of intergroup comparison for healthy lung vs. AP.

**Table 2:** Results of histogram analysis and HU threshold-derived HAA/rHAA.

	NECT					CTPA				
	Healthy Lung		AP		*p*	Healthy Lung		AP		*p*
	Mean	SD	Mean	SD		Mean	SD	Mean	SD	
**STD**	128.50	6.51	170.16	39.09	<0.001	134.43	10.39	160.90	32.23	<0.001
**SKEW**	3.50	0.35	2.36	0.90	<0.001	2.85	0.50	2.33	0.70	<0.001
**KURT**	14.29	2.83	6.70	4.93	<0.001	9.76	3.26	6.45	3.76	<0.001
**MEAN**	−828.00	28.54	−751.69	89.79	<0.001	−770.49	48.99	−750.68	76.14	0.185
**MEDIAN**	−863.64	27.00	−807.24	91.90	<0.001	−808.73	47.77	−801.84	67.74	0.842
**Lung Volume**	4973.00	1212.19	4302.19	1363.27	<0.001	4238.08	1185.40	4390.15	1322.73	0.400
**HAA −600/0**	266.37	49.43	574.18	468.63	<0.001	349.82	101.22	547.60	318.80	<0.001
**HAA −600/−250**	197.94	38.06	401.22	236.25	<0.001	278.10	89.04	416.63	231.34	<0.001
**HAA −700/−251**	347.83	79.54	674.60	336.70	<0.001	572.42	250.36	753.26	368.96	<0.001
**HAA −800/−500**	721.20	302.14	1148.24	473.73	<0.001	1457.43	601.75	1412.97	539.84	0.655
**rHAA −600/0**	5.57	1.48	9.12	4.78	<0.001	15.01	11.93	14.03	10.03	<0.001
**rHAA −600/−250**	4.15	1.22	7.31	4.22	<0.001	10.63	7.74	10.66	7.33	<0.001
**rHAA −700/−251**	7.39	3.29	15.48	11.04	<0.001	17.95	11.92	19.60	13.53	0.002
**rHAA −800/−500**	15.74	9.42	38.57	21.18	<0.001	30.16	16.50	35.70	18.30	0.465

Abbreviations: AP = atypical pneumonia. NECT = non-enhanced computed tomography. CTPA = computed tomography pulmonary angiogram. STD = histogram standard deviation. SKEW = skewness. KURT = kurtosis. MEAN = mean lung attenuation in HU. MEDIAN = median lung attenuation in HU. HAA = high attenuation area in mL. rHAA = relative high attenuation area in percentage. Lung volume in mL. SD = standard deviation. *p* = *p*-value of intergroup comparison for healthy lung vs. AP.

**Table 3:** Potential of imaging biomarkers to separate control vs. AP in NECT.

Parameter	MIC
**STD**	0.374
**rHAA −600/0**	0.346
**HAA −600/−250**	0.325
**SKEW**	0.311
**HAA −600/0**	0.307
**KURT**	0.307
**rHAA −600/−250**	0.295
**HAA −700/−251**	0.272
**rHAA −700/−251**	0.257
**MEAN**	0.195
**MEDIAN**	0.168
**rHAA −800/−500**	0.163
**HAA −800/−500**	0.155
**Lung volume**	0.059

MIC ranges from 0 to 1 and measures the degree of dependency between the variables, whereas 0 indicates no dependency and higher values indicate higher dependency. Concretely, a MIC of 0 means a random distribution of the given parameter in both groups (normal and AP). Therefore, this parameter would not be suited to assign an examination to one of these groups. The concept is based on entropy estimations from k-nearest neighbors’ distances. Abbreviations: AP = atypical pneumonia. NECT = non-enhanced computed tomography. MIC = Mutual Information Classifier. STD = histogram standard deviation. SKEW = skewness. KURT = kurtosis. MEAN = mean lung attenuation. MEDIAN = median lung attenuation. HAA = high attenuation area. rHAA = relative high attenuation area.

## Data Availability

All data supporting the reported results will be uploaded on a publicly available repository, with no identifiable patient information. The methods used are available here: https://github.com/usb-radiology. A sample dataset of 20 CTs underlying this article with associated demographic information is freely available in the following repository [hyperlink https://www.rapmed.net/#/publications/NECT-CTPA]. Please refer to Supplement S5 for further details. All software and code used in this study are available online and without restriction. The algorithm for lung segmentation and reporting tool can be found as a link in the original publication [11]. The Python libraries used are open-access and can be found following the links: SciPy [45]: https://www.scipy.org/scipylib/download.html. pandas [48]: https://pandas.pydata.org/. scikit-learn [49]: https://scikit-learn.org/stable/install.html. matplotlib [50]: https://matplotlib.org/users/installing.html. seaborn [51]: https://seaborn.pydata.org/installing.html.

## Supplementary Materials

The following are available online at https://www.mdpi.com/article/10.3390/diagnostics11050738/s1, Supplementary documents S1–S4 provide further details on parameters and results.

## Abbreviations

AP	Atypical Pneumonia
CI	Confidence Interval
COVID-19	Corona Virus Disease 2019
CRP	C-Reactive Protein
CTPA	CT Pulmonary Angiogram
GGO	Ground Glass Opacity
HAA	High Attenuation Area (absolute)
HU	Hounsfield Unit
IRB	Institutional Review Board
KURT	Kurtosis
MEAN	Mean Lung Attenuation
MEDIAN	Median Lung Attenuation
MIC	Mutual Information Classifier
ML	Machine Learning
NECT	Non-Enhanced Computed Tomography
PACS	Picture Archiving and Communication System
rHAA	High Attenuation Area (relative)
RT-PCR	Reverse Transcription Polymerase Chain Reaction
SARS-CoV-2	Severe Acute Respiratory Syndrome Coronavirus 2
SD	Standard Deviation
SKEW	Skewness
STD	Histogram Standard Deviation
WHO	World Health Organization
