# Supplementary material for: Automated CT Lung Density Analysis of Viral Pneumonia and Healthy Lungs Using Deep Learning-Based Segmentation, Histograms and HU Thresholds
Source: Diagnostics (Basel). 2021 Apr 21;11(5):738. doi: 10.3390/diagnostics11050738 (PMC8143124; doi:10.3390/diagnostics11050738)
Supplement: Supplementary file 1 [file diagnostics-11-00738-s001.zip › diagnostics-1179711-supplementary.pdf]

Supplement

Supplement 1: CT protocol, details

Mean peak tube voltage was 103.04 kVp (standard deviation [SD] 13.45), mean pitch factor 1.65 (SD 0.72), mean tube current-time product 292.79 mAs (SD 745.68 mAs). Mean computer tomography dose index was 4.15 mGy (SD 3.46 mGy), mean dose length product 161.48 mGy/cm (SD 159.61 mGy/cm). For CTPAs, an average of 72.2 ml (SD: 24.4) of contrast agent was applied (Iopamiro 370, Bracco Suisse SA; Ultravist 370, Bayer AG; or Xenetix 350, Guerbet AG).

Supplement 2: Histogram analysis parameters, details

**Abbreviation**	Parameter	Description
**STD**	Histogram Standard Deviation	Standard deviation within the histogram, a measure of the spread of a distribution.
**SKEW**	Skewness	Skewness is a measure of asymmetry. A histogram that is shifted from a normal histogram shape to the right converges further to gaussian normal distribution which leads to a decrease of the skewness value towards zero.
**KURT**	Kurtosis	Measure of peakness. For a histogram exceeding the gaussian normal distribution, kurtosis values rise, for a histogram flattening, kurtosis values diminish.
**MEAN**	Mean Lung Attenuation	Sum of the density of all voxels divided by the amount of voxels
**MEDIAN**	Median Lung Attenuation	HU with 50% of the voxels being of higher HU and 50% being of lower HU

Supplement 3: Modified WHO Scale for Clinical Severity

Modified WHO Scale for Clinical Severity [44] in AP as used in this analysis. AP = atypical Pneumonia.

Patient State	Descriptor	Score
outpatient	limitation of activity	1
hospitalized - Mild disease	hospitalized, no oxygen therapy	2
	oxygen by mask or nasal prongs	3
hospitalized - severe disease	non-invasive ventilation or high-flow oxygen	4
	intubation and mechanical ventilation	5
	ventilation + additional organ support - pressor, RRT, ECMO	6

Supplement 4 R-squared of correlations of imaging biomarkers with clinical parameters

Coefficient of determination (r-squared) of correlations (lower triangle) and 95% confidence intervals (upper triangle, with lower confidence interval in first row and upper in second row, respectively) of imaging biomarkers with clinical parameters (CRP and clinical severity scale). The colormap applies to the Spearman rank correlation values and the range of confidence intervals. Abbreviations: NECT = non-enhanced computed tomography. STD = histogram standard deviation. SKEW = skewness. KURT = kurtosis. MEAN = mean lung attenuation. MEDIAN = median lung attenuation. HAA = high attenuation area. rHAA = relative high attenuation area.

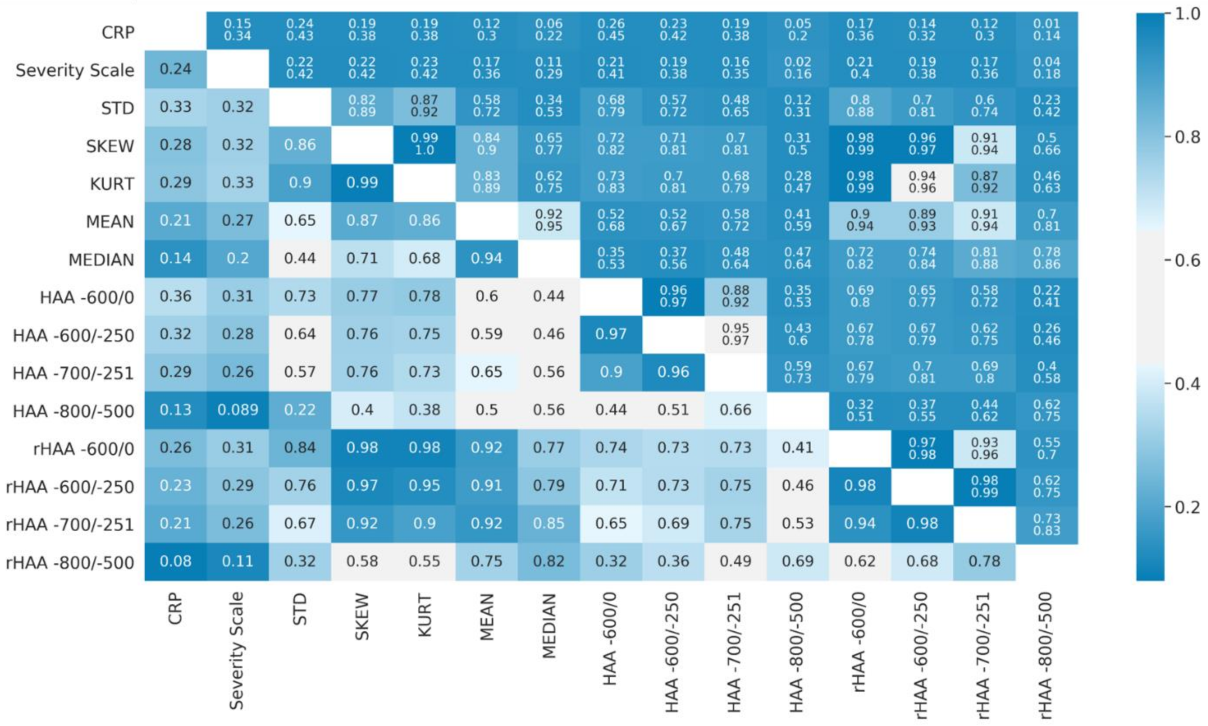

Supplement 5: Direct access to image data

List of 20 chest CTs analysed during this study. The complete 1 mm series in soft-tissue kernel reconstruction are available online on the RapMed imaging platform.

To access the data, please go to: https://www.rapmed.net/#/publications/NECT-CTPA

Other CT dataset are available from the corresponding author on reasonable requests.

Case ID	Group	RT-PCR result	Age	Sex
Case 1	0	0	33	2
Case 2	0	0	49	2
Case 3	0	0	25	1
Case 4	0	0	69	1
Case 5	0	0	72	2
Case 6	0	0	37	2
Case 7	0	0	82	1
Case 8	0	0	22	2
Case 9	0	0	52	1
Case 10	0	0	59	2
Case 11	1	1	46	1
Case 12	1	1	42	2
Case 13	1	1	56	2
Case 14	1	2	51	1
Case 15	1	2	65	2
Case 16	1	1	36	2
Case 17	1	2	75	1
Case 18	1	2	64	1
Case 19	1	2	52	1
Case 20	1	2	54	2

Group: 0 = healthy lung group; 1 = Atypical pneumonia group;

RT-PCR result: 0 = negative; 1 = positive for Influenza; 2 = positive for SARS-CoV-2;

Sex: 1 = male; 2 = female;
